# Association between Hypertension and Stroke Recurrence as Modified by Pro-oxidant–Antioxidant Balance: A Multi-Center Study

**DOI:** 10.3390/nu15102305

**Published:** 2023-05-14

**Authors:** Thu T. M. Pham, Tuyen Van Duong, Lien T. K. Nguyen, Manh-Tan Vu, Khue M. Pham, Minh H. Nguyen, Thuc C. Luong, Binh N. Do, Lan T. H. Le, Nga H. Dang, Thao T. P. Nguyen, Hoang P. Le, Cuong Q. Tran, Kien T. Nguyen, Chaur-Jong Hu, Chang-Chuan Chan, Hui-Chuan Hsu, Chyi-Huey Bai

**Affiliations:** 1 School of Public Health, College of Public Health, Taipei Medical University, Taipei 110-31, Taiwan; phamminhthu.ytcc@gmail.com (T.T.M.P.); gingerhsu@tmu.edu.tw (H.-C.H.); 2 Faculty of Public Health, Hai Phong University of Medicine and Pharmacy, Hai Phong 042-12, Vietnam; pmkhue@hpmu.edu.vn; 3 School of Nutrition and Health Sciences, Taipei Medical University, Taipei 110-31, Taiwan; 4 Rehabilitation Department, Hanoi Medical University, Hanoi 115-20, Vietnam; lienrehab@hmu.edu.vn; 5 Rehabilitation Center, Bach Mai Hospital, Hanoi 115-19, Vietnam; 6 Rehabilitation Department, Viet Duc University Hospital, Hanoi 110-17, Vietnam; 7 Department of Internal Medicine, Haiphong University of Medicine and Pharmacy, Hai Phong 042-12, Vietnam; vmtan@hpmu.edu.vn; 8 Cardiovascular Department, Viet Tiep Friendship Hospital, Hai Phong 047-08, Vietnam; 9 President Office, Hai Phong University of Medicine and Pharmacy, Hai Phong 042-12, Vietnam; 10 School of Preventive Medicine and Public Health, Hanoi Medical University, Hanoi 121-08, Vietnam; drminh.ttytlc@gmail.com; 11 Director Office, Military Hospital 103, Hanoi 121-08, Vietnam; lcthuc@gmail.com; 12 Department of Cardiology, Cardiovascular Center, Military Hospital 103, Hanoi 121-08, Vietnam; 13 Department of Infectious Diseases, Vietnam Military Medical University, Hanoi 121-08, Vietnam; nhubinh.do@vmmu.edu.vn; 14 Division of Military Science, Military Hospital 103, Hanoi 121-08, Vietnam; 15 Training and Direction of Healthcare Activity Center, Thai Nguyen National Hospital, Thai Nguyen City 241-24, Vietnam; lanhuong.bvtutn@gmail.com (L.T.H.L.); danghoangngatn@gmail.com (N.H.D.); 16 Biochemistry Department, Thai Nguyen National Hospital, Thai Nguyen City 241-24, Vietnam; 17 Director Office, Thai Nguyen National Hospital, Thai Nguyen City 241-24, Vietnam; 18 Department of Quality Control, Thai Nguyen National Hospital, Thai Nguyen City 241-24, Vietnam; 19 Institute for Community Health Research, University of Medicine and Pharmacy, Hue University, Hue 491-20, Vietnam; nguyenthiphuongthao@hueuni.edu.vn; 20 Department of Internal Medicine, University of Medicine and Pharmacy, Hue University, Hue 491-20, Vietnam; lephuochoang@hueuni.edu.vn; 21 Director Office, Thu Duc City Health Center, Ho Chi Minh City 713-10, Vietnam; quoccuong.mph@gmail.com; 22 Faculty of Health, Mekong University, Vinh Long 852-16, Vietnam; 23 Department of Health Promotion, Faculty of Social and Behavioral Sciences, Hanoi University of Public Health, Hanoi 119-10, Vietnam; ntk1@huph.edu.vn; 24 Department of Neurology, School of Medicine, College of Medicine, Taipei Medical University, Taipei 110-31, Taiwan; chaurjongh@tmu.edu.tw; 25 Department of Neurology, Taipei Medical University Shuang Ho Hospital, New Taipei City 235-61, Taiwan; 26 Institute of Environmental and Occupational Health Sciences, College of Public Health, National Taiwan University, Taipei 100-55, Taiwan; ccchan8082@gmail.com; 27 Innovation and Policy Center for Population Health and Sustainable Environment (Population Health Research Center, PHRC), College of Public Health, National Taiwan University, Taipei 100-55, Taiwan; 28 Global Health Program, College of Public Health, National Taiwan University, Taipei 100-55, Taiwan

**Keywords:** stroke recurrence, hypertension, oxidative stress, pro-oxidant–antioxidant balance, health behavior

## Abstract

Background: Hypertension and oxidative stress are involved in the pathophysiological mechanism of stroke. We aimed to investigate the modification impact of the pro-oxidant–anti-oxidant balance (PAB) on the association between hypertension and stroke recurrence (SR). Methods: A cross-sectional design was conducted from December 2019 to December 2020 in 951 stroke patients in six hospitals across Vietnam. Hypertension was defined using antihypertensive medication or systolic blood pressure ≥ 140 mmHg or diastolic blood pressure ≥ 90 mmHg. PAB was estimated using weighting methods based on smoking, drinking, and overweight/obesity with pro-oxidant capacity, diet quality, fruit intake, vegetable intake, and physical activity with antioxidant capacity. The higher PAB scores indicated a beneficial balance shifting toward antioxidant dominance. SR was diagnosed by neurologists. Moreover, sociodemographic and health conditions were included as covariates. Multiple logistic regression analyses were used to explore the associations and interactions. Results: The hypertension and SR proportions were 72.8% and 17.5%, respectively. hypertension was associated with an increased SR likelihood (odds ratio (OR) = 1.93; *p* = 0.004), whereas a higher PAB score was associated with a lowered SR likelihood (OR = 0.87; *p* = 0.003). Moreover, hypertension interacting with every one-point increment of PAB was associated with a lowered SR likelihood (OR = 0.83; *p* = 0.022). Conclusions: The harmful impact of hypertension on SR could be alleviated by PAB. The interplay of health behaviors should be highlighted in the intervention strategies for stroke prevention.

## 1. Introduction

To date, stroke remains the most common cause of disability and mortality world-wide, which leads to a substantial global health burden [1]. The highest rate of stroke incidence was found in Southeast Asian countries, including Vietnam [2]. Although multilevel and multisectoral prevention strategies have been applied, the stroke recurrence (SR) rate has not changed over the years [3,4]. Moreover, the risk of SR increases over time [5], causing continuously increasing issues in societies and medical systems around the world [6], as well as in Vietnam [7]. Therefore, closing the gap in stroke prevention strategies is a high priority. 

About 90% of strokes are caused by the presence of modifiable risk factors, and the regulation of core health behaviors (such as diet, physical activity, weight, and smoking) could avert about 75% of this burden [6,8]. Hypertension (HTN) is the most frequent danger for stroke and recurrent events [9,10]. Although hypertensive management is the most important primary and secondary prevention strategy for stroke, managing blood pressure in stroke patients is complex and challenging [11]. Moreover, evidence-based prevention strategies are not obviously improving modifiable risk factors and recurrent cardiovascular events (including stroke) [12]. Therefore, exploring factors that could be accessible for modifying the harmful impact of HTN on SR is necessary for prevention strategies. 

Oxidative stress (OS) is characterized by the pro-oxidant–antioxidant balance (PAB) shifting toward the pro-oxidant predominance, resulting in molecular damage [13,14,15]. The existing evidence showed that the OS response could derive from endogenous (e.g., metabolic processes) and exogenous sources (e.g., core health behaviors), which are involved in the pathological mechanism of chronic diseases [16], including stroke [17,18,19]. In the endogenous aspect, OS is linked closely to chronic inflammatory disorders, which were identified as the major triggers for cardiovascular diseases (CVDs). The close interaction between OS and inflammatory response was reported regarding the overexpression of reactive oxygen species (ROS)-producing enzymes (e.g., NADPH NOXs) or aggravated inflammatory phenotypes in the absence of antioxidant defense proteins (e.g., glutathione peroxidases, heme oxygenase-1, and superoxide dismutase) [20]. Moreover, OS plays a critical role in the mechanism of CVDs through endothelial dysfunction, of which oxidative enzyme systems (e.g., xanthine oxidase and NADPH oxidase) contribute to the inactivation of nitric oxide—an endothelial regulator, leading to endothelial dysfunction [21]. In the exogenous aspect, potential diets and nutraceuticals could reduce OS and display antioxidant effects, which represents a therapeutic target in CVDs [21]. Additionally, regulating PAB could be a promising strategy for stroke therapeutics [22].

The PAB can be approached from pro-oxidative and antioxidative perspectives, and both can be considered in terms of their individual chemical constituents or their pooled effect [23]. Pro-oxidants can be from intracellular sources (such as NADPH NOXs, peroxisomes, and mitochondria) and external sources (such as pollutants, ultraviolet light, and ionizing) [24], whereas endogenous antioxidants involve the products of cellular metabolism (e.g., peroxiredoxin, catalase, and superoxide dismutase), and exogenous antioxidants involve diets and medications [24]. In the literature, the PAB has been directly estimated in saliva, urine, plasma, and serum [23], demonstrating the contribution of exogenous and endogenous agents. However, for example, serum PAB cannot reflect each agent’s specific pro-oxidant and antioxidant potentials and the complex interaction between the agents. Therefore, an indirect measurement tool based on lifestyle, nutrition, and medication factors was developed and validated to evaluate the individual PAB status and its impact on health outcomes [25,26,27]. However, the evidence regarding the indirect estimation of PAB based on health behaviors impacting SR is limited. Therefore, we aimed to examine the relationship between health behavior-based PAB and SR. Further, the modification role of PAB in the association between HTN and SR was explored.

## 2. Materials and Methods

### 2.1. Study Population

A cross-sectional study was launched from December 2019 to December 2020 to enroll patients with stroke in Vietnam. Due to the COVID-19 crisis, six invited hospitals agreed to participate in our study. Stroke patients were identified by neurologists using the 10th revision of the International Classification of Disease (ICD-10) coding I60–I69 [28], who were recruited from rehabilitation, neurology, and cardiovascular departments. Patients with stable stroke conditions (e.g., a Mini-Mental State Examination score of ≥22, vital signs within normal limits, recovery after the acute stroke and hospitalized at least one month), aged ≥ 18 years, and being able to reply to questions were eligible for selection. Moreover, we excluded patients with aphasia and impairment of vision and cognition. A minimum sample of 715 was estimated using SAS v9.4 software based on eight predictors in multiple logistic regression with a power of 0.8 and an alpha of 0.1. A satisfactory sample of 951 qualified patients was recruited for the final analysis. This study was reviewed and approved by the Institutional Ethical Review Committee of the Hanoi School of Public Health, Vietnam (IRB No. 498/2019/YTCC-HD3 and No. 312/2020/YTCC-HD3).

### 2.2. Data Collection and Measurement

#### 2.2.1. Data Collection Procedure

A four-hour training session regarding data collection and infection control (e.g., wearing a mask, washing hands, and physical distancing) during the COVID-19 pandemic was provided for the interviewers (including medical students, doctors, and nurses) according to the guidelines provided by the World Health Organization (WHO) [29] and the Ministry of Health in Vietnam [30]. Then, patients were asked to sign an informed consent form before participating in a 30 min face-to-face survey at the bedside of each patient.

#### 2.2.2. Assessment of Outcome

Stroke recurrence (SR) was identified by asking patients and their families whether the current hospitalization was the first or recurrent (including the number of times) stroke. Then, patients were regrouped into the first stroke vs. recurrent stroke to facilitate the analysis because of the limited sample size for the number of times of recurrent events.

#### 2.2.3. Assessment of Hypertension

A standard clinical manual aneroid sphygmomanometer was used for three-time measurements of systolic blood pressure (SBP) and diastolic blood pressure (DBP). The average SBP and DBP were computed. Additionally, participants’ information about taking antihypertensive medication was obtained. Hypertension was identified as using any antihypertensive medication, SBP ≥ 140 mmHg, or DBP ≥ 90 mmHg [31,32].

#### 2.2.4. Assessment of Pro-oxidant–Antioxidant Balance

The PAB was indirectly established in the literature based on multiple components and comparable weighting methods (such as equal-weighted, study data-based, literature review-derived, and Bayesian methods) [33,34]. In the current study, the PAB components were defined based on core health behaviors related to stroke, including overweight/obesity, smoking, and drinking with pro-oxidant properties, physical activity, diet quality, fruit intake, and vegetable intake with antioxidant properties.

- Drinking frequency and smoking status were self-reported. Body mass index (BMI, kg/m^2^) was computed and classified into under/normal weight, overweight, and obesity [35,36].
- Diet quality was assessed using the Dietary Approaches to Stop Hypertension Quality (DASH-Q) questionnaire [37], and the 10-item DASH-Q questionnaire was validated for use in the Vietnamese context [38]. Patients were asked how many days they ate the food items over the previous seven days. A DASH-Q score was summed (ranging between 0 and 70) and categorized into tertiles, with greater tertiles indicating healthier diet quality.
- Fruit intake and vegetable intake were assessed using the STEPwise questionnaire with four typical questions that patients were asked about their consumption of fruit and vegetables in terms of the number of days per week and the number of servings per day. The WHO STEPwise questionnaire was validated in the Vietnamese context [39]. According to WHO recommendations adopted in Asian countries (including Vietnam) [40], a minimum daily intake of five servings of fruit and vegetables (including two fruit and three vegetable servings) was proposed to prevent chronic diseases.
- Physical activity was assessed using a short version of the International Physical Activity Questionnaire (IPAQ) [41], which was validated for use in the Vietnamese context [42,43]. Patients were asked how many minutes per day and days per week over the previous seven days they undertook sitting, walking, and moderate and vigorous activities. Then, the sum score of minutes per week for each of those four activities was multiplied by 1, 3.3, 4, and 8, respectively, to estimate the equivalent metabolic task scored in minutes per week (MET/min-wk) [44]. A higher MET score indicated a more intensive PA level. According to WHO [45] and the Physical Activity Guidelines Advisory Committee [46], a minimum PA level of 600 to 1200 MET/min-wk is recommended for adults with chronic conditions or disabilities.

All components were categorized into three levels and assigned 0, 1, or 2 values, accordingly. Then, the PAB score was calculated by summing all components after weighting by multiplying the values with −1 for pro-oxidant and +1 for antioxidant (Table 1). The PAB score ranged from −6 to 8, with higher PAB scores indicating a beneficial balance shifting toward antioxidant dominance.

#### 2.2.5. Assessment of Covariates

Socio-demographic factors (including age, gender, educational attainment, occupation, social status, marital status, and ability to pay for medication) were self-reported.

Stroke classification was categorized based on the ICD-10 into infraction (including cerebral infarction (I63)), hemorrhage stroke (including subarachnoid hemorrhage (I60), intracerebral hemorrhage (I61), other nontraumatic intracranial hemorrhage (I62)), and others (including stroke not specified as hemorrhage or infarction (I64); occlusion and stenosis of precerebral arteries, not resulting in cerebral infarction (I65); occlusion and stenosis of cerebral arteries, not resulting in cerebral infarction (I66); other cerebrovascular diseases (I67); cerebrovascular disorders in diseases classified elsewhere (I68); and sequelae of cerebrovascular disease (I69)).

Comorbidity was judged using the 16-item Charlson comorbidity index (CCI), which is validated and used widely in Vietnam [47,48]. In the current study, items of cerebrovascular disease or stroke and dementia were not included in the comorbid conditions due to the study participants and exclusion criteria, respectively. Additionally, the item of depression was not counted as a comorbidity to avoid the duplicated assessment. The remaining items were regrouped into none vs. one/more CCI to simplify the analysis.

Depressive symptoms were assessed using a two-item patient health questionnaire (PHQ-2), which was suggested for use in busy medical settings [49,50]. Patients were asked about the frequency of being affected by positive or depressed moods in the past two weeks and were rated on a three-point scale from 0 (not at all) to 3 (nearly every day). The PHQ-2 score ranged from 0 to 6, and a score ≥ 2 was suggested for clinicians to ensure that the few cases of depression were not overlooked [51].

Health literacy (HL) (including three domains of healthcare, disease prevention, and health promotion) was estimated using a 12-item short-form survey (HLS-SF12). The difficulty of performing each item was rated from 1 (very difficult) to 4 (very easy) based on a 4-point Likert scale. The HL index (the standardized HL indices) was calculated using the following formula:

$$\mathrm{Index}=\frac{\left(mean-1\right)\times 50}{3}$$

in which *mean* displays the average of 12 items, while *1*, *3*, and *50* display the minimal possibility of the mean, the range of the mean, and the chosen maximum HL index score, respectively. The HL index varied from 0 (worse HL) to 50 (best HL).

### 2.3. Statistical Analysis

First, descriptive analyses were performed, and the independent sample T-test (or Kruskal–Wallis test) and chi-square test were used for continuous and categorical variables, respectively. Second, logistic regression analysis was used to examine the relationship between HTN and PAB with SR. The independent variables (IVs) with *p* < 0.2 in the bivariate analysis were selected for the multiple regression models. The multicollinearity among those IVs was controlled by examining their correlation using Spearman’s correlation. Because moderate correlations were found between age and occupation (*rho* = 0.34) and between gender and PAB (*rho* = −0.35) (Table S1), the representative variables were selected for multiple analysis models, including age, education, ability to pay for medication, stroke classification, depressive symptoms, and CCI. Third, interaction analysis was used to explore the combined impact of HTN and PAB on SR. Furthermore, the results of the interaction model were visualized via a simple slope analysis using PROCESS Macro of SPSS for moderation analysis. The slope plots were drawn using the estimated values of SR probability for two categories of HTN (yes vs. no) by three levels of PAB (one standard deviation below the mean (−1SD), the mean, and one standard deviation above the mean (+1SD)). All analyses were conducted using SPSS version 22 (IBM Corp., Armonk, NY, USA), and the *p*-value < 0.05 was defined as a statistically significant result.

## 3. Results

### 3.1. Characteristics of Stroke Patients

Out of 951 patients with stroke, the proportions of SR and HTN were 17.5% and 72.8%, respectively. The age ranged from 19 to 99 years, and the proportion of patients aged ≥65 was 53.7%. Compared to participants with the first stroke, those with SR had a higher percentage of age ≥ 65 (*p* = 0.011), were men (*p* = 0.042), were retired or infirm (*p* = 0.021), and had an infarction stroke (*p* = 0.012), HTN (*p* < 0.001), and CCI (*p* < 0.001) (Table 2).

### 3.2. Determinants of Stroke Recurrence

Among the investigated factors, the multiple analysis results (Table 3) showed that HTN (odds ratio, OR, 1.93; 95% confidence interval, 95%CI, 1.23, 3.04; *p* = 0.004) and CCI (OR, 1.55; 95%CI; 1.08, 2.23; *p* = 0.017) were associated with a higher likelihood of SR. Whereas PAB was associated with a lower likelihood of SR (OR, 0.87; 95%CI, 0.80, 0.95; *p* = 0.003). Moreover, hemorrhage stroke had a lower likelihood of recurrence compared to infarction stroke (OR, 0.55; 95%CI, 0.34, 0.90; *p* = 0.019).

### 3.3. Modification Impact of PAB on the Association between HTN and Stroke Recurrence

**Figure 1:**
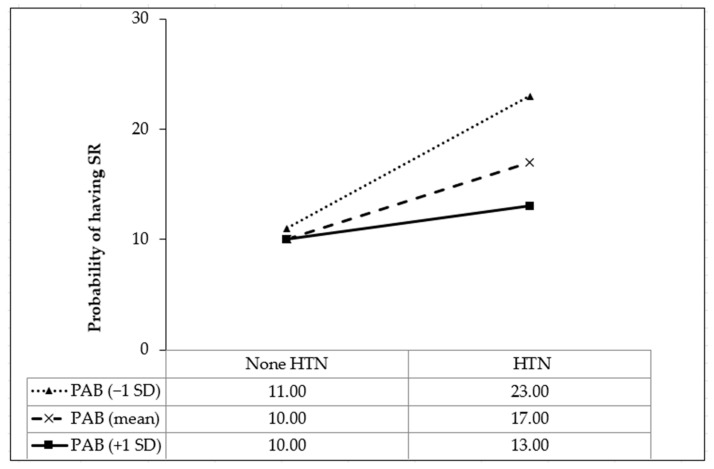
Simple slope plot for the interaction between hypertension and pro-oxidant–antioxidant balance on stroke recurrence (*n* = 951). HTN, hypertension; PAB, pro-oxidant–antioxidant balance; SD, standard deviation.

As shown in Table 4, compared to stroke patients without HTN and with the lowest PAB scores, those with HTN had an increased likelihood of SR (OR, 2.06; 95%CI, 1.28, 3.32; *p* = 0.003). However, the likelihood of SR decreased in stroke patients with HTN and with every one-point increment of PAB (OR, 0.83; 95%CI, 0.66, 0.94; *p* = 0.022). Moreover, the results of interaction were visualized in Figure 1. Simple slope analysis showed that the negative impact of HTN on SR was lowered by higher PAB values from −1SD (OR, 2.43; 95%CI, 1.29. 4.57; *p* = 0.005) to the mean (OR, 1.82; 95%CI, 1.16, 2.88; *p* = 0.008) and +1SD (OR, 1.36; 95%CI, 1.13, 2.58; *p* = 0.003).

## 4. Discussion

The current findings emphasize the positive impact of PAB on lowering SR, especially regarding the moderating role of PAB in mitigating the negative impact of HNT on SR.

Although OS is involved in the pathological mechanism of stroke, the study of OS in stroke patients is challenging to undertake because of the complexly interrelated processes. Further, OS is caused by numerous exogenous factors, and the combined (either addictive or synergistic) influences of those factors on the OS process should be considered. Therefore, a global estimation of the PAB score was established based on various dietary, lifestyle, and medication components [25]. In the literature, a low PAB score (reflecting excessive OS) was associated with poor health outcomes (such as chronic kidney diseases and all-cause mortality) [25], but little is known about PAB score and SR. Previous studies directly estimated the plasma PAB concentration and found heterogeneity associations between PAB and SR. Although the redox unbalances were greater in patients within one month of stroke onset than in their controls [52], the serum PAB concentration at the acute stroke phase was not associated with SR within five years [53]. To our knowledge, the current study was the first to find that a higher health behavior-based PAB score reflecting antioxidant properties could reduce the likelihood of SR.

The relationship between OS and inflammation is a vicious circle that links to HTN [54]. In stroke patients, persistent inflammatory responses are activated in those with HTN, which leads to an additional chain of OS procedures, and the HTN-OS correlation contributes to increasing SR [55]. That is why eliminating the free radical agents in the OS processes via “scavengers” is able to decrease the adverse consequences of stroke [55]. Previous studies showed that although the association between the antioxidative and anti-inflammatory capacities of a single dietary component with the hypotensive impact was not clear, adding such a component may become a new method for controlling the balance in OS–inflammation crosstalk [56]. Our findings supported the rationale that a greater PAB score could minimize the deleterious influence of HTN on SR probability. In the current study, the PAB score was estimated based on diet quality, fruit and vegetable intake, and physical activity in regard to anti-oxidative capacity. Therefore, the interplay of those components should be taken into consideration for the balanced regulation of OS and inflammation, as well as for preventing SR.

In recent decades, dietary nutrients have been used a novel intervention for building cardiovascular and neurological health due to their antioxidant properties [57]. However, the mechanism regarding the relationship between diet nutrients and neuro-cardiological diseases is not yet well defined, that metabolic control (including lipid, ROS, and energy metabolisms), immunological regulation (including astrocyte and microglial activation), and epigenetic modification (including non-coding RNA, DNA methylation, and histone modification) were assumed to be involved. Regarding dietary nutrients, the intervention programs focused on two forms of diet, including dietary supplements (such as vitamins and plant extracts) and dietary restriction (such as the DASH diet with decreasing cholesterol, total fat, and saturated fat and increasing fibers and minerals). Moreover, fruits and vegetables are rich sources of vitamins and plant extracts containing polyphenolic components, which are potent antioxidants and are reported to be beneficial to cardiovascular health [58]. However, the excessive intake of antioxidative dietary nutrients (e.g., iron and vitamin A) could lead to the side effects, such as cell death, because of ROS overproduction, causing OS. Additionally, there are anti-nutrient compounds contained in fruits and vegetables (such as tannins, oxalates, and lectins), which may threaten health [59]. Therefore, the approach of PAB needs to consider both the individual pro-oxidant and antioxidant components and their mutual effects.

The present study is the first to assess the potential role of PAB score in altering the relationship between HTN and SR. However, several limitations should be noted. First, the associations were recognized, but the causal inference could not be established in a cross-sectional study. Second, as the study participants were recruited in stable conditions of stroke, the findings could apply only to mild and moderate stroke patients but not to those with severe stroke conditions. However, the sample size was satisfactory for representativeness, with a power of evidence of up to 90%. Third, the questionnaires were responded to via self-reporting, and the “standard serving” size of food was not defined, which may lead to underestimation.

## 5. Conclusions

The current findings highlighted the critical contribution of PAB to prevent SR and mitigate the negative impact of hypertension on SR. Because multiple factors contribute to oxidative stress, we suggest that health promotion programs should include multiple dimensions to promote an overall healthy lifestyle to reduce oxidative stress. In the future research, the interplay of health behaviors should be considered in the strategic intervention of balancing the oxidative stress–inflammation relationship and preventing SR.

## Figures and Tables

**Table 1:** Categories and weights of pro-oxidant–antioxidant balance components.

Component	Categories	Definition	Weights
Pro-oxidants			
Smoking	Never	Never smoked	0
	Used to	Former smoker	−1
	Current	Currently smokes	−2
Drinking	None	Never drink	0
	Moderate	Drink 1–4 times/month on average	−1
	Heavy	Drink daily or at least ≥ 3 times/week	−2
BMI (kg/m^2^)	Under/normal weight	BMI < 23	0
	Overweight/obesity	23 ≤ BMI < 27.5	−1
	Obesity	BMI ≥ 27.5	−2
Anti-oxidants			
Fruit intake	Low	<2 servings/day	0
	Moderate	2 servings/day	+1
	High	>2 servings/day	+2
Vegetable intake	Low	<3 servings/day	0
	Moderate	3 servings/day	+1
	High	>3 servings/day	+2
Diet quality	Low	DASH-Q ≤ 24	0
	Moderate	24 < DASH-Q ≤ 35	+1
	High	DASH-Q > 35	+2
Physical activity (MET-min/wk)	Low	MET < 600	0
	Moderate	600 ≤ MET ≤ 1200	+1
	High	MET > 1200	+2

**Table 2:** Characteristics and stroke recurrence (*n* = 951).

Variables	Total	First Stroke	Recurrent Stroke	
	*n* (%)	(*n* = 785, 82.5%)	(*n* = 166, 17.5%)	*p*
Socio-demographics				
Age (years)				0.011
<65	440 (46.3)	378 (48.2)	62 (37.3)	
≥65	511 (53.7)	407 (51.8)	104 (62.7)	
Gender				0.042
Woman	388 (40.8)	332 (42.3)	56 (33.7)	
Man	563 (59.2)	453 (57.7)	110 (66.3)	
Occupation				0.021
Working	518 (54.5)	441 (56.2)	77 (46.4)	
Retirement or infirmity	433 (45.5)	344 (43.8)	89 (53.6)	
Education attainment				0.363
Illiterate or elementary	216 (22.7)	181 (23.1)	34 (20.5)	
Junior high school	257 (27.1)	204 (26.0)	53 (31.9)	
Senior high school	251 (26.4)	206 (26.3)	45 (27.1)	
College/university or higher	227 (23.9)	193 (24.6)	34 (20.5)	
Ability to pay for medication				0.129
Very or fairly difficult	423 (44.5)	358 (45.6)	65 (39.2)	
Very or fairly easy	528 (55.5)	427 (54.4)	101 (60.8)	
Marital status				0.979
Married	837 (88.0)	691 (88.0)	146 (88.0)	
Single or Widowed/Divorced/Separated	114 (12.0)	94 (12.0)	20 (12.0)	
Social status				0.335
Low	111 (11.7)	88 (11.2)	23 (13.9)	
Middle or high	840 (88.3)	697 (88.8)	143 (86.1)	
HL index (mean ± SD)	23.4 ± 10.0	23.5 ± 10.0	22.8 ± 9.9	0.426
Health conditions				
Stroke classification				0.012
Hemorrhage	220 (23.2)	196 (25.0)	24 (14.5)	
Infraction	637 (67.1)	511 (65.3)	126 (75.9)	
Others	92 (9.7)	76 (9.7)	16 (9.6)	
Depressive symptoms				0.145
No	461 (48.5)	372 (47.4)	89 (53.6)	
Yes	490 (51.5)	413 (52.6)	77 (46.4)	
CCI				<0.001
No	476 (50.1)	415 (52.9)	61 (36.7)	
Yes	475 (49.9)	370 (47.1)	105 (63.3)	
Hypertension				<0.001
No	259 (27.2)	232 (29.6)	27 (16.3)	
Yes	692 (72.8)	553 (70.4)	139 (83.7)	
PAB (median, IQR)	1.0 (0.0, 3.0)	1.0 (0.0, 3.0)	0.0 (−1.0, 2.0)	<0.001

Abbreviation: HL, health literacy; SD, standard deviation; CCI, Charlson comorbidity index; PAB, pro-oxidant–antioxidant balance; IQR, interquartile range.

**Table 3:** Determinants of stroke recurrence (*n* = 951).

Variables	Stroke Recurrence					
	Crude Model			Adjusted Model		
	OR *	95%CI *	p *	OR **	95%CI **	p **
Socio-demographics						
Age (years)						
<65	1.00			1.00		
≥65	1.55	1.10–2.19	0.012	1.28	0.88–1.85	0.191
Gender						
Woman	1.00					
Man	1.44	1.01–2.04	0.042			
Occupation						
Working	1.00					
Retirement or infirmity	1.48	1.05–2.07	0.022			
Education attainment						
Illiterate or elementary	1.00			1.00		
Junior high school	1.38	0.86–2.22	0.181	1.22	0.74–2.01	0.420
Senior high school	1.16	0.71–1.89	0.544	1.17	0.70–1.95	0.546
College/university or higher	0.93	0.55–1.57	0.808	0.87	0.50–1.52	0.638
Ability to pay for medication						
Very or fairly difficult	1.00			1.00		
Very or fairly easy	1.30	0.92–1.83	0.129	1.18	0.82–1.71	0.361
Marital status						
Married	1.00					
Single or Widowed/Divorced/Separated	1.01	0.60–1.68	0.979			
Social status						
Low	1.00					
Middle or high	0.78	0.47–1.28	0.336			
HL index	0.99	0.97–1.01	0.426			
Health conditions						
Stroke classification						
Hemorrhage	1.00			1.00		
Infraction	2.01	1.26–3.21	0.003	1.79	1.10–2.91	0.019
Others	1.71	0.86–3.41	0.121	1.68	0.82–3.43	0.152
Depressive symptoms						
No	1.00			1.00		
Yes	0.77	0.55–1.09	0.145	0.79	0.56–1.12	0.194
CCI						
No	1.00			1.00		
Yes	1.93	1.36–2.72	<0.001	1.55	1.08–2.23	0.017
Hypertension						
No	1.00			1.00		
Yes	2.16	1.39–3.35	0.001	1.93	1.23–3.04	0.004
PAB	0.84	0.77–0.91	<0.001	0.87	0.80–0.95	0.003

Abbreviation: OR, odds ratio; CI, confidence interval; HL, health literacy; CCI, Charlson comorbidity index; PAB, pro-oxidant–antioxidant balance. * Results of bivariate logistic regression analysis. ** Results of multivariate logistic regression analysis adjusted for age, education, ability to pay for medication, stroke classification, depressive symptoms, and CCI.

**Table 4:** Interaction of hypertension and pro-oxidant–antioxidant balance on stroke recurrence (*n* = 951).

Interaction	Stroke Recurrence					
	Crude Model			Adjusted Model		
	OR *	95%CI *	*p* *	OR **	95%CI **	*p* **
Non-HTN × PAB (lowest score)	1.00			1.00		
HTN × PAB (lowest score)	2.12	1.33–3.38	0.002	2.06	1.28–3.32	0.003
Non-HTN × PAB (1-point increment)	0.95	0.78–1.16	0.677	0.90	0.82–1.23	0.934
HTN × PAB (1-point increment)	0.86	0.69–0.97	0.012	0.83	0.66–0.94	0.022

Abbreviation: OR, odds ratio; CI, confidence interval; HTN, hypertension; PAB, pro-oxidant–antioxidant balance. * Results of bivariate logistic regression analysis. ** Results of multivariate logistic regression analysis, adjusted for age, education, ability to pay for medication, stroke classification, depressive symptoms, and comorbid conditions.

## Data Availability

The raw data supporting the findings of this article will be made available upon reasonable request to the corresponding authors.

## Supplementary Materials

The following supporting information can be downloaded at: https://www.mdpi.com/article/10.3390/nu15102305/s1, Table S1: Spearman’s correlation (*rho*) among the studied variables (*n* = 951).
