# Supplementary material for: Association between Hypertension and Stroke Recurrence as Modified by Pro-oxidant–Antioxidant Balance: A Multi-Center Study
Source: Nutrients. 2023 May 14;15(10):2305. doi: 10.3390/nu15102305 (PMC10224087; doi:10.3390/nu15102305)
Supplement: Supplementary file 1 [file nutrients-15-02305-s001.zip › nutrients-2367716-supplementary.pdf]

Table S1. Spearman's correlations (*rho*) among the studied variables (n= 951).

Variables	Age	Gender	Occupation	Education attainment	Ability to pay for medication	Stroke classification	Depressive symptoms	HTN	Comorbid condition
Gender	-0.08								
Occupation	**0.34**	-0.01							
Education attainment	-0.21	0.23	0.08						
Ability to pay for medication	-0.05	0.02	0.14	0.25					
Stroke classification	-0.16	0.01	-0.09	0.05	-0.02				
Depressive symptoms	-0.02	-0.03	-0.03	0.01	-0.01	0.00			
HTN	0.10	-0.00	0.13	-0.02	0.03	-0.02	-0.01		
Comorbid condition	0.07	-0.01	0.11	0.05	0.15	-0.09	-0.00	0.11	
PAB	-0.02	**-0.35**	-0.13	-0.04	-0.09	0.04	0.00	-0.13	-0.18

Abbreviation: HTN, hypertension; PAB, prooxidant-antioxidant balance.
